# Chronic Ketamine Toxicity Involving both Urinary and Hepatobiliary Systems

**DOI:** 10.5334/jbsr.4197

**Published:** 2026-01-20

**Authors:** Alexander Cotza, Liesbeth Meylaerts

**Affiliations:** 1 Department of Radiology, Ziekenhuis Oost-Limburg, Genk, Belgium

**Keywords:** ketamine abuse, ketamine toxicity, ketamine-induced uropathy, ketamine-induced cholangiopathy

## Abstract

Chronic ketamine abuse is a well-recognized cause of lower urinary tract injury. Hepatobiliary manifestations are less frequently reported. A case is presented of concomitant ketamine-induced uropathy and cholangiopathy in a young adult. This underscores the importance of considering ketamine toxicity as a multisystem disorder.

*Teaching point:* Beyond the well-recognized urinary tract involvement, radiologists attention is drawn to hepatobiliary manifestations of chronic ketamine toxicity.

## Introduction

Chronic ketamine abuse is known to harm the urinary tract, but its hepatobiliary effects are less well known. A case is described of a young adult with concurrent ketamine-induced uropathy and cholangiopathy.

## Case Report

A 22-year-old man with two years of daily ketamine use presented with lethargy, flank discomfort, and urinary symptoms of incontinence, pollakiuria, urgency, and pyuria. On examination, he was febrile (38.9 °C) with suprapubic and mild costovertebral angle tenderness. Laboratory tests revealed a markedly elevated CRP (241 mg/L) and impaired renal function (eGFR 20 mL/min/1.73 m², creatinine 3.91 mg/dL). Liver enzymes were diffusely abnormal. Leukocytosis was not present. Serologic tests for viral, HIV, and autoimmune causes were negative. Urine culture showed no bacterial growth.

**Figure 1:**
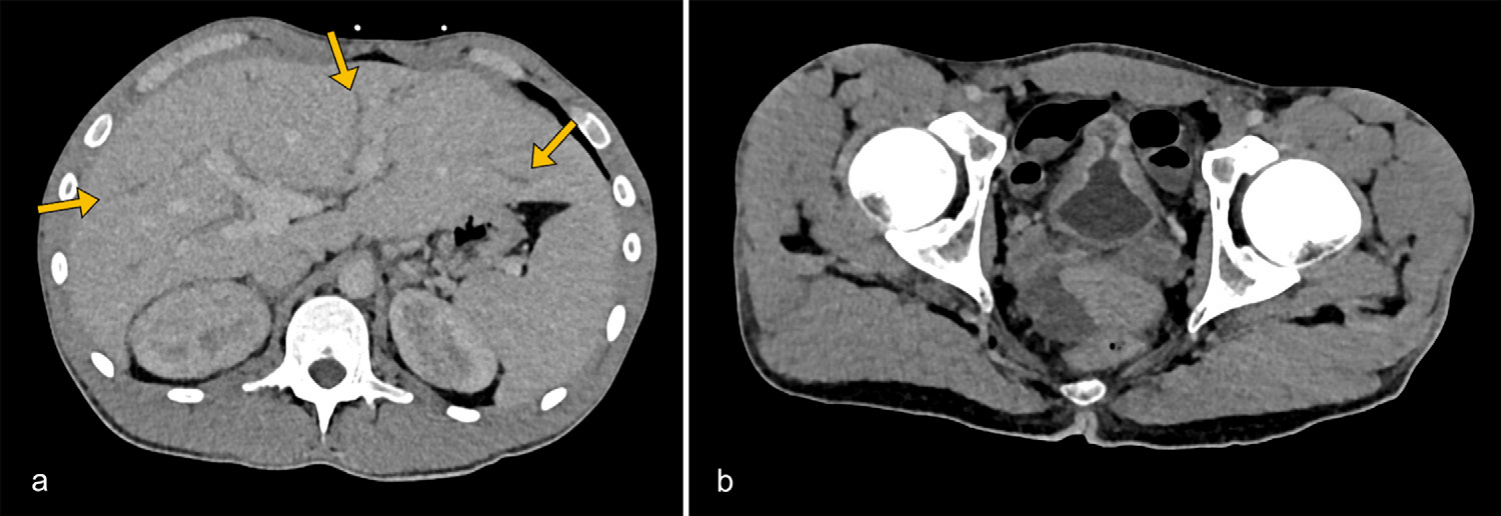
Contrast-enhanced axial CT images in the venous phase. **(a)** Hepatomegaly with prominent intrahepatic bile ducts (arrows). **(b)** Thickened and enhancing bladder wall with perivesical fat stranding, consistent with inflammatory cystitis. Symmetric mural thickening of the mid-portion causes luminal narrowing, producing an ‘hourglass’ configuration.

**Figure 2:**
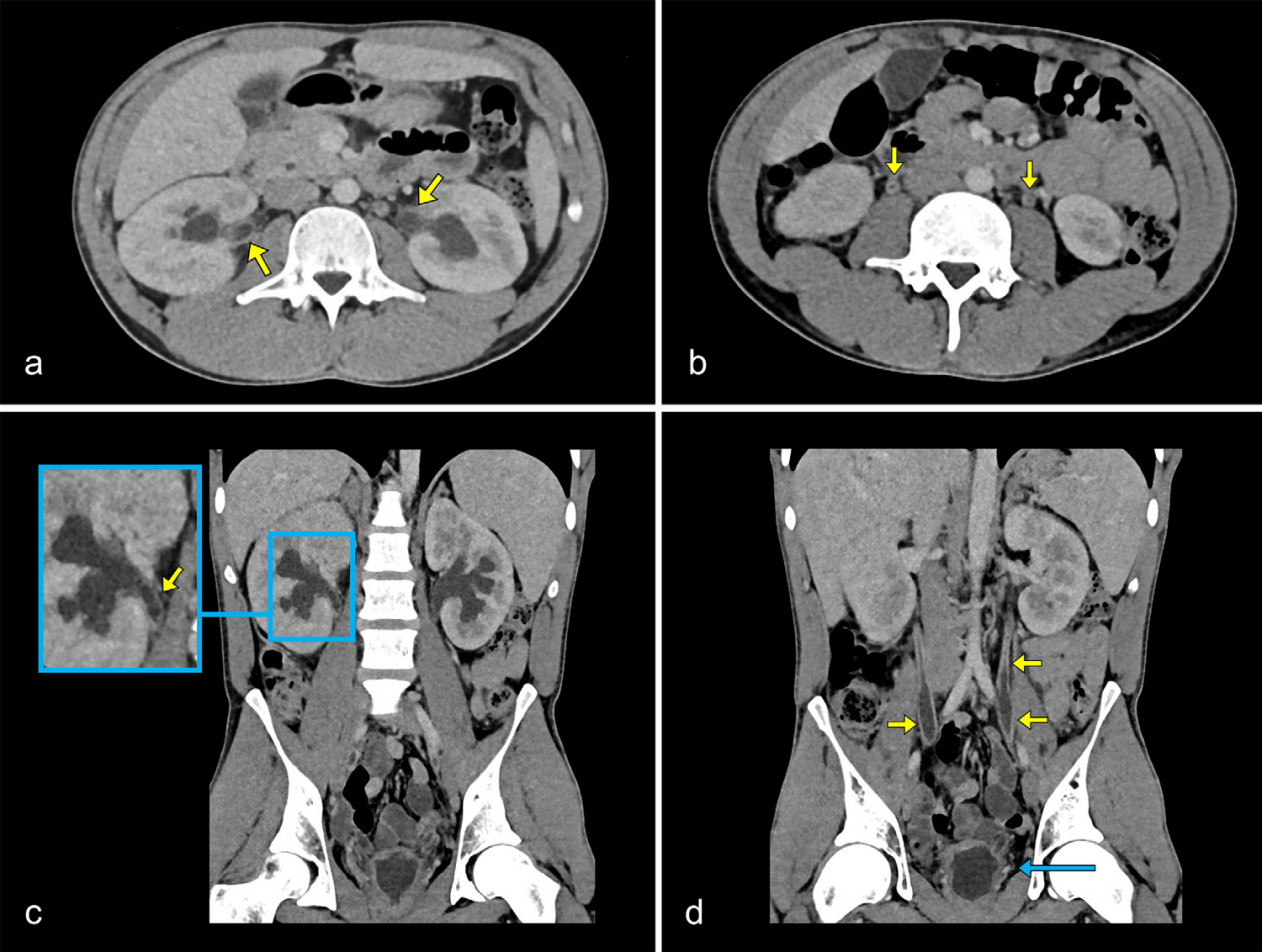
Contrast-enhanced axial **(a, b)** and coronal **(c, d)** CT images in the venous phase. Both kidneys appear bulky with delayed nephrograms and reduced corticomedullary differentiation. The walls of the renal pelvis and ureters are thickened and demonstrate marked contrast enhancement (yellow arrows). The bladder appears small and compressed with a markedly hypertrophic wall, indicating reduced capacity (blue arrow).

Contrast-enhanced CT (portal venous phase) revealed hepatomegaly with intrahepatic bile duct dilatation (Figure 1a). A shrunken, thick-walled bladder with perivesical fat stranding was present, consistent with inflammatory cystitis, and correlating with the patient’s urinary incontinence and urgency (Figure 1b). The bladder’s mid-portion was constricted with a characteristic ‘hourglass’ configuration. The kidneys appeared bulky with delayed and heterogeneous nephrogram. The excretory system was dilated symmetrically, with mural thickening and increased enhancement of the urothelium of the renal pelvis and ureters (Figure 2).

**Figure 3:**
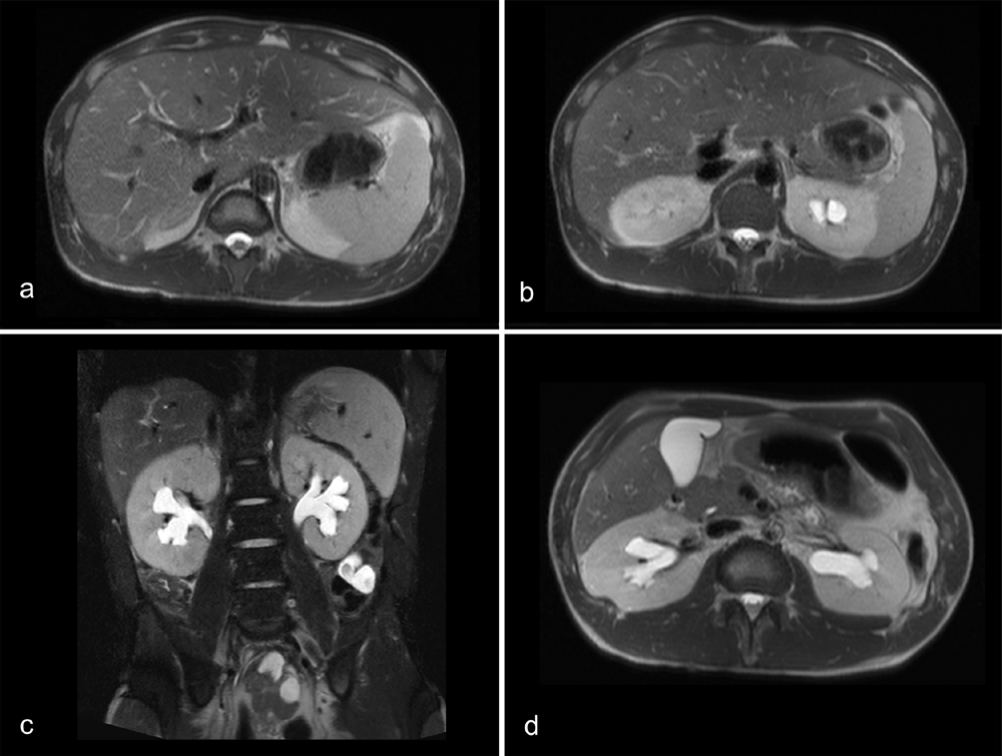
Axial **(a, b, d)** and coronal **(c)** T2-weighted HASTE fat-suppressed MR images showing hepatomegaly with prominent intrahepatic bile ducts and bilaterally enlarged, bulky kidneys.

**Figure 4:**
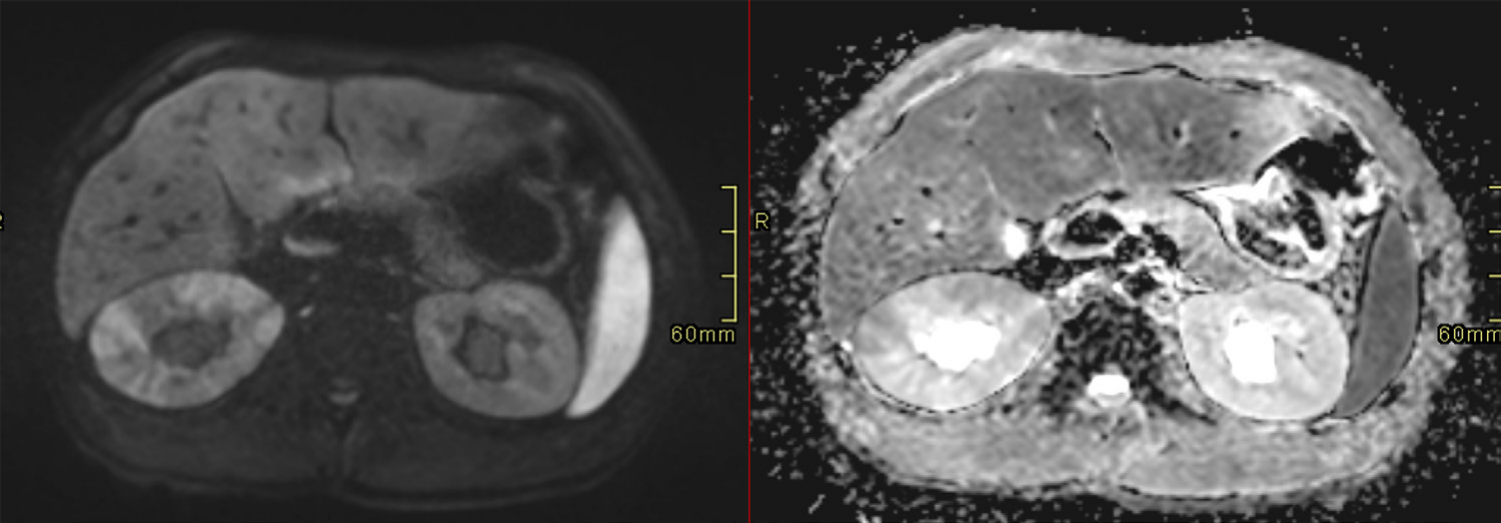
Axial diffusion-weighted (DWI) image (left) and corresponding apparent diffusion coefficient (ADC) map (right) showing restricted diffusion within the renal parenchyma, consistent with interstitial nephropathy.

**Figure 5:**
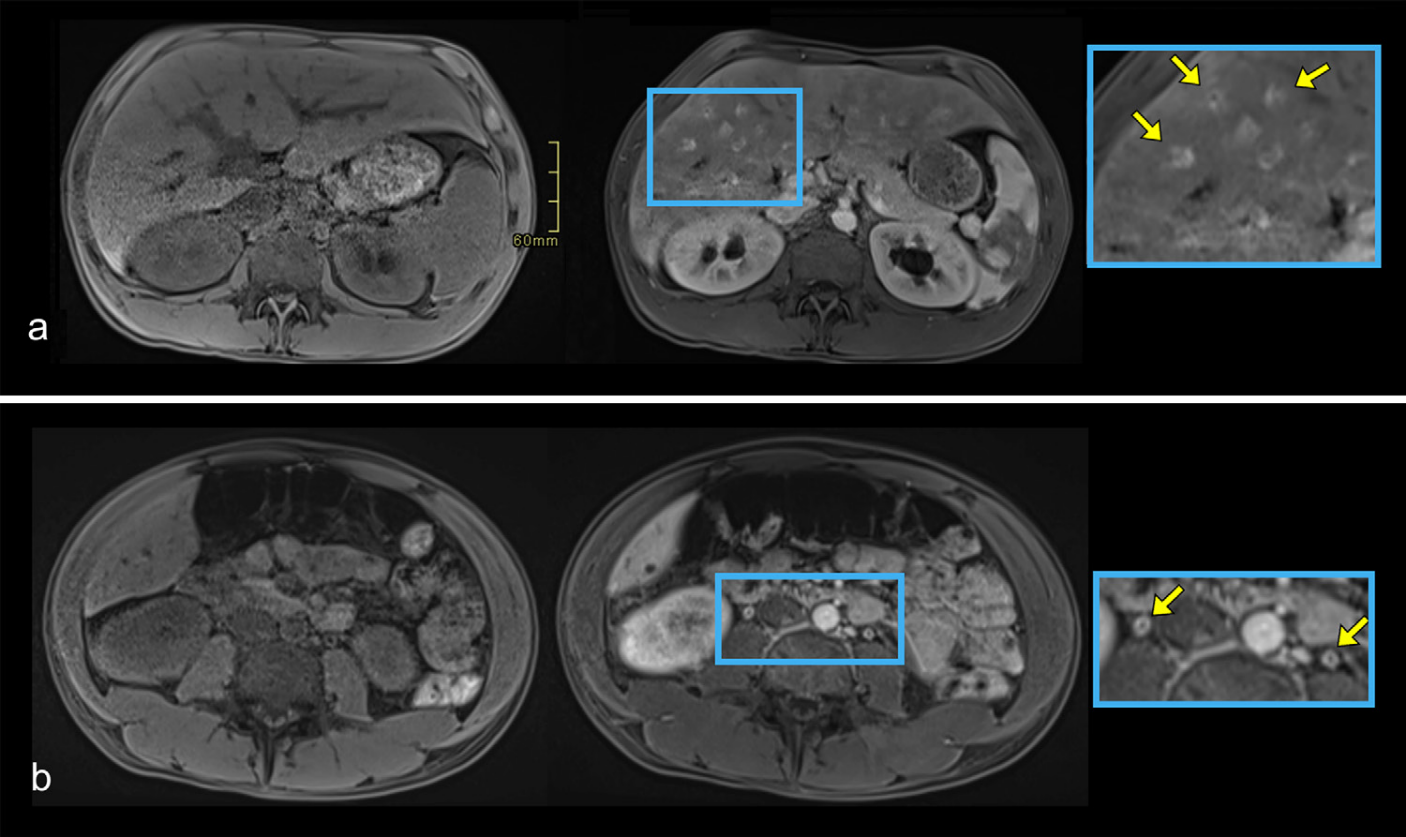
Axial T1-weighted MR images acquired before and during the arterial post-contrast phase demonstrate mural enhancement of the intrahepatic bile duct walls **(a)** and ureteral walls **(b)**, consistent with inflammatory cholangitis and ureteritis, respectively. Enlarged views in the blue boxes highlight the areas of mural enhancement. Yellow arrows indicate the enhancing walls.

To further assess the hepatobiliary involvement, an abdominal MRI was performed one week later. Inflammatory markers and renal function had improved while liver tests remained diffusely abnormal. MRI demonstrated persistent hepatomegaly with mild intrahepatic ductal dilatation, bilateral hydronephrosis, and bulky kidneys (Figure 3). Diffusion-weighted imaging showed patchy renal parenchymal diffusion restriction consistent with interstitial nephropathy (Figure 4). Arterial phase post-contrast images revealed marked mural enhancement of the intrahepatic bile duct and ureters, consistent with inflammatory cholangitis and ureteritis (Figure 5).

Overall, the imaging findings were consistent with ketamine-induced cholangiopathy and uropathy. Cessation of ketamine use, conservative management with fluids, antibiotics, and ursodeoxycholic acid resulted in normalization of CRP and recovery of liver and renal function within one week.

## Discussion

Ketamine is a dissociative anesthetic increasingly misused for its hallucinogenic effects. Chronic exposure can cause toxicity in both the urinary and hepatobiliary systems.

The drug is hepatically metabolized and mainly excreted renally. Bile duct injury likely results from epithelial toxicity and smooth muscle dysfunction, causing biliary stasis and inflammation [1–4].

In the urinary tract, ketamine causes urothelial inflammation and fibrosis, resulting in a low-capacity bladder with vesicoureteral reflux and hydronephrosis [5–7]. Patients typically present with severe lower urinary tract symptoms.

From a radiologic perspective, ketamine-induced injury poses a growing diagnostic challenge. Cross-sectional imaging largely contributes to assessing the extent of involvement and excluding other causes of obstruction, such as tumors or calculi [5].

In the hepatobiliary system, ketamine-induced cholangiopathy may present with dilatation of the extrahepatic bile duct, which can be diffuse but more typically is fusiform with distal tapering. The intrahepatic ducts may also be affected, with ductal dilatation, as in this case, as well as beading or strictures [1, 2, 8]. Mural thickening and enhancement of the bile ducts are characteristic findings, reflecting inflammatory involvement in the biliary wall [1, 2].

In the urinary tract, ketamine-induced uropathy typically presents with mural thickening of the bladder and ureters, accompanied by perivesical fat stranding and mucosal enhancement of the bladder and ureters. Mural thickening may lead to secondary luminal narrowing, and along with the reduced bladder capacity, hydroureteronephrosis is frequently observed [5–7]. The so-called ‘hourglass bladder’ results from focal, often symmetrical, mural thickening in the mid-portion of the bladder, creating two communicating compartments. Once considered congenital, this configuration has also been described in neurogenic, tuberculous, and post-surgical cystitis, and has also been reported in chronic ketamine-induced cystitis, although it is not a consistent finding [9].

Recognition of these characteristic imaging findings, in conjunction with clinical and laboratory data, is particularly helpful for diagnosis and helps avoid unnecessary invasive tests. Although there is no established treatment, cessation of ketamine use is essential. Ursodeoxycholic acid or, in selected cases, biliary stenting may be considered for cholangiopathy [2, 4].

## Conclusion

Ketamine-induced toxicity represents an emerging diagnostic entity with a broad imaging spectrum. While urinary tract involvement is well documented, hepatobiliary injury remains somewhat underrecognized. Growing awareness of its imaging features contributes to an accurate diagnosis and appropriate management.
